# Improving the kinematic accuracy of a collaborative continuum robot by using flexure-hinges

**DOI:** 10.1016/j.heliyon.2024.e26144

**Published:** 2024-02-10

**Authors:** N. Ma, D. Cheneler, S.D. Monk

**Affiliations:** Department of Engineering, Lancaster University, Lancaster, United Kingdom

**Keywords:** Continuum robot, Dual-arm cooperation, Underwater sample retrieval, Parallel mechanism

## Abstract

Within various unstructured industrial environments, there is often the requirement to conduct remote engineering tasks, such as sampling the structure for analysis prior to decommissioning. Most existing tools are simply not dexterous enough to fulfil this task, and thus new technology is required. We describe here a simple, lightweight, and water-resistant collaborative dual-arm continuum robot system which can aid in this task. To improve the kinematic accuracy of the system, a class of flexible hinges have been combined with a conventional continuum robot configuration. The thickness and width of said flexible hinges can be adjusted to adapt to various tasks. Kinematic and stiffness models have further been developed, incorporating the influence of these flexible hinges. A set of experiments have been conducted to validate the proposed model and demonstrate the advantages of the platform. It was found that the kinematic accuracy of the continuum robot can be improved by a factor of around 10 with the aid of said hinges.

## 1 Introduction

One of numerous challenges within the nuclear industry is operating on objects submerged under contaminated water [1,2]. There are over 400 civil nuclear reactors in operation globally, which require periodic inspection and repair, and eventual decommissioning [3,4]. One of the major decommissioning challenges is the clean-up of radioactive material to a safe point before demolition of the facilities. This is a hazardous, expensive, and time-consuming process when the environment is underwater. Higher accuracy can be achieved by introducing commercial robotic arms (e.g., UR) but the bulk dimension is normally the challenge. The existing self-developed tools are not dexterous enough or not strong enough for these tasks and it is hard to adapt them to bespoke tasks [5,6]. The objective of this study is to develop a novel dual-manipulator continuum robot system with improved kinematic accuracy and stiffness for use in unstructured industrial environments, particularly underwater confined spaces. This system exhibits improved kinematic accuracy and stiffness and is optimised for use within unstructured industrial environments, with a focus on underwater confined spaces. Following on from the previous research in building the stiffness model [32] and developing a collaborative controller [33], this paper focusses on the study of the benefits of adding the flexure hinges to the platform.

Most underwater manipulators available for in-situ environments are developed for oil tanker [7] and ship inspection [8], with conventional arm structures generally favoured [9,10]. A unified 6-DoF lightweight robotic arm has been developed for confined spaces by Yin et al. [11], although built-in actuators at the joints ensure the Centre Of Mass (COM) is far from the base, causing significant instability. Further, a 7-DoF lightweight robotic arm, has been constructed for the subsea service industry by Koch et al. [12], although it is too bulky to be combined with a commercial ROV and used within a confined space as desired. Recently, an ROV, constructed with two 6-DoF arm robots, was developed for subsea intervention [13], although this system was primarily designed for heavy-duty conditions where space is not a concern and thus the platform is unsuitable for confined environments. In addition to pure bulk, the actuators are close to the joints of the arms, causing instability. Generally, there are limited options available in terms of small and highly dexterous underwater manipulators for confined spaces.

In order to circumnavigate these issues, continuum robots are thus used here. In this configuration, all the actuators are located within an actuation pack attached to a centrally located ROV system and connected to continuum manipulators via driving cables, thus offering enhanced stability. Another important advantage of these manipulator types is the reduction in required dimension of the system [[14], [15], [16]]. A long length continuum robot (1.2 m long, 26-DoF) was developed for the inspection of an engine via a small inspection port (diameter: 12.9 mm) [[17], [18], [19]], though the stiffness was too low for general inspection applications. A multi-section, continuous-backbone continuum robot actuated by pressured air, has been prototyped to physically adapt with the environment [20], although obviously air compressors are bulky and heavy and thus this type of device would not be useful in the target application considered here. Further, a kind of hybrid continuum robot, which is actuated by the combination of cables and pneumatic muscles, has been developed to regulate stiffness for use within safety operations [21]. Besides the industrial applications, continuum robots are further popular within clinical environments [[22], [23], [24]]. For example, a super-elastic NiTi - based continuum robot has been developed to perform minimally invasive surgery [25], where the gripper and camera end-effectors are delivered to the desired position via a small entrance port. However, the kinematic accuracy is typically too poor to perform the tasks with any great accuracy.

The cable based flexible mechanism proposed here, features relatively low stiffness compliant components [26], enabling the transfer of force and displacement to another point through the elastic body deformation. This brings advantages, such as small form factor [27] and adjustable stiffness [28]; ideal if combined with the continuum robot principle to improve kinematic performance. Numerous small and flexible mechanisms have been developed which feature leaf springs allowing linear motion and the replacement of conventional prismatic joints [29]. Further, a compliant parallel universal joint, constructed with cross-spring pivots, was developed to achieve a constant rotation stiffness during bending [30]. Besides that, a large-range decoupled XY compliant parallel manipulator, which can achieve orthogonal motion within a plane, has been developed to perform micro-positioning ability [31]. However, the aforementioned compliant mechanisms were only designed to complete a single function (i.e. rotational or linear movement).

In order to reduce the bulk while improving the kinematic accuracy of the continuum robot, a novel compliant mechanism involving compliant hinges is combined here with a conventional continuum robot configuration. Further, the stiffness model of the proposed hybrid dual-arm continuum robot has been established, which considers the influence of the compliant hinges. Finally, after experimentally validating the proposed models with the prototyped setups, performance of the system was tested to demonstrate the benefits of introducing the flexure hinge into the continuum robot system. The summarized objectives can be seen in the following:

(i) Each section of the continuum robot must have 2-DoF bending motion, with axial rotation minimized.
(ii) The centre of mass of the system must be within the stability region of the parent ROV.
(iii) The stiffness of the two collaborative arms must be sufficient to maintain operational accuracy.
(iv) All electrical units must be within the actuation pack in order to ensure waterproofing.

The first point of novelty in this work is the combination of a compliant mechanism with a continuum robot to improve the kinematic performance of the conventional backbone-structure and high length-diameter ratio manipulators. Based on this idea, a class of novel compliant mechanisms have been developed (e.g., flexible hinges, compliant rods and hyper-elastic soft materials) to facilitate the adaption to different environments. The second point of novelty concerns the new stiffness model of the proposed 6-DoF continuum robot, incorporating the models of the modular cable-driven 2-DoF parallel mechanism and the novel flexible hinges. By introducing the compliant mechanism into the continuum robot, the mechanical performance (e.g., adjustable stiffness, reduced backlash) can be largely controlled for coping different challenges. In next step, different kinds of compliant mechanisms (e.g., flexible hinges with different structure, hyperplastic material with different shape) will be incorporated into the continuum robots to improve their performance. Further, the new kinetostatic model and collaborative operation strategy will be developed with the consideration of the influence of the compliant mechanisms.

## 2 System description

### 2.1 Structure of continuum robot with flexible hinges

**Fig. 1:**
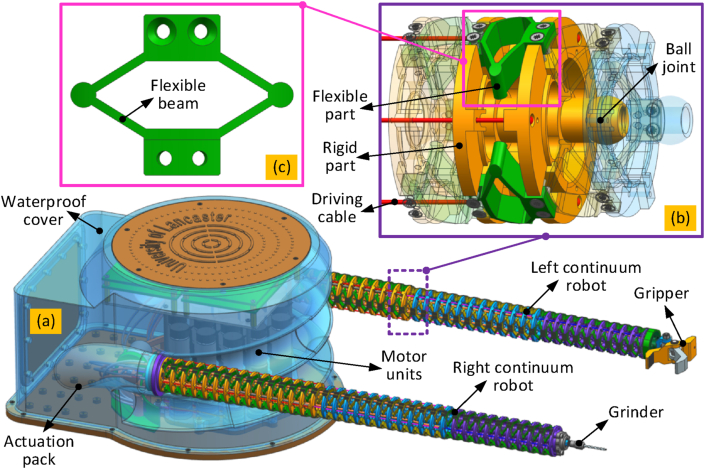
The dual continuum robot with flexure-hinges: (a) is the overall structure of the platform with two extended compliant continuum manipulators; (b) is the structure of the disk with three equally distributed compliant hinges; (c) is the example structure of the compliant hinge.

To ensure the centre of mass (COM) is within the balance region of the ROV, the actuating motors of the robot and end-effectors are located in the actuation pack located under the ROV. To achieve this, a cable driving method has been adopted in order to transfer actuation to the manipulators as shown in Fig. 1(a). In addition, to improve the kinematic accuracy of the continuum robot, a decoupled actuation strategy was adopted ensuring independent motion of each section. As the cable driving method is adopted to actuate the shape variation of the sections, the driving cables for the fore sections (i.e., close to the tip) need to pass through the base sections. With the decoupled actuation strategy, to actuate the bending of fore sections, the flexible spring tubes were adopted to transmit the power of motor directly, no longer need to pass through the based sections. In this During operation, the gripper fitted manipulator adjusts its configuration in terms of position and orientation to ensure a firm grip. Then, the milling manipulator runs the planned trajectory to cut the material from the environment as required.

Each 2-DoF section of the continuum robot needs to achieve orthogonal rotational motion. To achieve the desired behaviour, each 2-DoF segment within the section includes three identical leaf spring-like flexure hinges that are equally radially spaced around central ball joints. As seen in Fig. 1, these flexure hinges have been designed to have a hexagonal shape with circular corners, to improve their load-bearing performance.

Up to now, to the best knowledge of the authors, most of the continuum robots that have incorporated additional compliant elements into its structure to enhance its stiffness have done so by modifying the compliance of the central shaft in the backbone. Whereas the inclusion of flexure hinges (i.e., hinges constructed with compliant mate-rial with specific mechanical properties) within the modular structure of a continuum robot to improve the kinematic accuracy, as well as the corresponding kinetostatic modelling to predict its performance, has not been utilized. Here, the enhanced performance of a continuum robot arm that incorporates novel leaf spring-like flexure hinges is demonstrated. The design of the hinges themselves as well as their contributions to the kinematic performance is discussed in this paper. The hinges were fabricated and integrated into a three-section 6-DoF prototype continuum robot and experimentally evaluated, thus demonstrating how the inclusion of flexure hinges significantly im-prove the stiffness, payload capacity and positional accuracy over conventional continuum robots.

Each of the manipulators constitutes three serially connected sections. In conventional continuum designs, the cables connecting with the fore section are passed through the rear sections. This strategy minimizes the outer diameter of the continuum robot, although it reduces the kinematic accuracy. Here, a novel decoupled actuation strategy was utilized. By changing the length of the driving cables connected to the motors located within the actuation pack, the manipulators can be bent into different shapes as required. With a central channel in each disk (as shown in Fig. 1(b)), the end-effector can be moved to the required position and orientation.

To accurately control the 2-DoF bending motion of each section, three driving cables are placed around the central ball joint as seen in Fig. 1(b). To improve the kinematic performance, the flexible diamond-shape leaf hinges (Fig. 1(c)) are further placed around the central ball joints. When a manipulator is bent by pulling the driving cables, the flexible hinges along the bending direction will be compressed, while the opposite flexible hinges will be stretched. These elastic deformations in the flexible hinges can improve the backlash and stability of the system. To enable the system to undertake tasks such as the removal of solid materials, a drilling adaptor was developed enabling the use of Dremel tools such as drills or cutting disks. To aid in this sample removal process, a gripper was developed and equipped at the tip of the other continuum manipulator.

### 2.2 Simulation validation of the concept

The stiffness of the flexible hinges determines the tension of the driving cables. If this is too high then greater tension will be induced in the driving cable, thus higher elongation will be generated in the cable, leading to a larger position deviation at the tip. In addition, in order to achieve higher kinematic accuracy, the rotational stiffness around the axial direction should be higher than the bending direction, as the rotational stiffness is uncontrollable in this kind of cable driving parallel system.

**Fig. 2:**
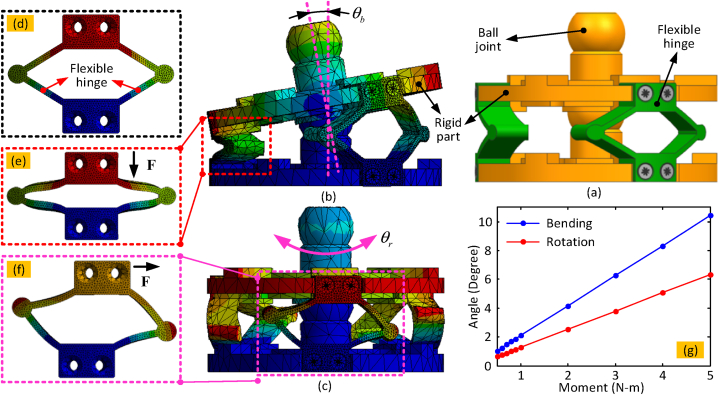
(a) and (b) are the simulated bending and rotational characters of the continuum snake, respectively; (c) is the structure of one segment; (d) is the stiffness character in bending and rotational direction under the given load; (e) to (g) are the deformation simulation of flexible hinge in compression and rotational directions, respectively.

In order to achieve the optimised performance of the long continuum arm, the parameters of the key structures (e.g., diameter of the arm, width, thickness and height of the flexible hinge) need to be selected carefully. As a case study, the deformation of a typical 2-DoF manipulator (Fig. 2(a)) was simulated using Finite Element Analysis (FEA - ANSYS, version 2017). The standard ABS material is selected as the example for this case study. During the simulation, the lower platform is fixed while the external load (e.g., moment) is applied at the upper platform to form the deformation. The base of the lower disk in the segment was fixed, and a moment was applied about the axis of rotation and an orthogonal axis of the upper disk to impose rotation and bending about the central ball joint (as seen in Fig. 2(b) and (c)). A flexible hinge was also modelled in isolation (Fig. 2(d)) to ascertain its deformation under compressive and shear loads (seen in Fig. 2(e) and (f) respectively) and thus its bending and rotational stiffness.

The diameter of the continuum arm was designed based on the application (e.g., hard material removal), where higher stiffness is required. The shape and parameters of the flexible hinges were designed based on the desired performance of the system (e.g., higher rotational stiffness and lower bending stiffness). With the wide research on the compliant mechanisms, the diamond shape was selected at last, which satisfies all the aforementioned requirements. It can be seen from Fig. 2(g) that the bending stiffness (0.49 N m/deg) of the diamond-shape structure of the flexible hinge is lower than the rotational stiffness (0.6 N m/deg), which is good for the higher kinematic performance.

## 3 Modelling of the compliant continuum robot

Here, three 2-DoF continuum sections are modelled. Unlike the conventional approach where only the geometrical parameters were considered for establishing the input-output of the system, a novel kinetostatic model was developed for improving the kinematic accuracy. This method considers the deformation forces of the flexible hinges, which were then utilized to forecast the tension of the driving cables. Thus, a more accurate and reliable kinematic calculation can be achieved.

**Fig. 3:**
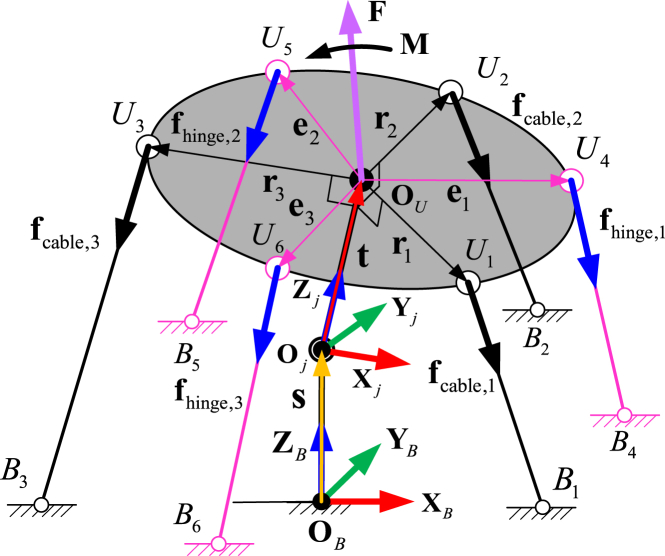
Schematic of the static configuration of the example 2-DoF segment.

The kinematic accuracy of the system is heavily influenced by the elastic deformation forces within the compliant hinges [34]. Thus, stiffness modelling was carried out in this section to study the overall stiffness characteristics of the continuum robot and the nonlinear stiffness behaviour of the compliant hinges. There are three main factors affecting this stiffness. First is the stiffness of the driving cables themselves (adjusted by regulating the tensions), second is the stiffness of the compliant hinges and third is the stiffness of the central shaft. The latter two are dependent on the configuration of the 2-DoF segment. When the upper platform moves to the required positions, the deformations of the compliant hinges, as well as the force in the central shaft, are different, thus affecting the stiffness character of the overall system. A schematic diagram of the static configuration of the example 2-DoF segment is shown in Fig. 3.

The static equation can be established by determining the force and moment equation at the ball joint point, which can be written as Eq. (1):

(1) $$\left\{ \begin{array}{c} \sum_{i=1}^{3}f_{\text{cable},i}+\sum_{i=1}^{3}f_{\text{hinge},i}+f_{s}+F=0\\ \sum_{i=1}^{3}\left(t+r_{i}\right)\times f_{\text{cable},i}+\sum_{i=1}^{3}\left(t+e_{i}\right)\times f_{\text{hinge},i}+M=0 \end{array}\right.$$

where $f_{\text{cable},i}$ is the tension force of the driving cable; $f_{\text{hinge},i}$ is the elastic deformation force of the compliant joint; $f_{s}$ is the compressing force of the central shaft; F and M are the external force and moment on the upper platform respectively, and $r_{i}$ is the position vector from the centre of the upper platform, $U_{i}$, to the *i*-th cable attaching point. Eq. (2) is composed from three items: the driving cables, the compliant hinges, and the central shaft. Thus, the stiffness matrix of the example 2-DoF segment can be written in the following format:

(2) $$K_{\text{segment}}=J_{\text{cable}}^{T}K_{\text{cable}}J_{\text{cable}}+J_{\text{hinge}}^{T}K_{\text{hinge}}J_{\text{hinge}}+J_{\text{joint}}^{T}K_{\text{joint}}J_{\text{joint}}$$

where $K_{\text{segment}}$ is the stiffness of the 2-DoF segment; $J_{\text{cable}}$, $J_{\text{hinge}}$ and $J_{\text{joint}}$ are the Jacobian matrices relating to the structural configuration of the 2-DoF segment; and $K_{\text{cable}}$, $K_{\text{hinge}}$ and $K_{\text{joint}}$ are the stiffness matrices of the driving cables, compliant hinges and central support, respectively. The stiffness matrix of the driving cables $K_{\text{cable}}$ can be expressed in the following format as Eq. (3):

(3) $$K_{\text{cable}}=\left[\begin{array}{ccc} k_{\text{cable},1} & 0 & 0\\ 0 & k_{\text{cable},2} & 0\\ 0 & 0 & k_{\text{cable},3} \end{array}\right]$$

where $k_{\text{cable},1}$, $k_{\text{cable},2}$ and $k_{\text{cable},3}$ are the stiffness parameters of the three driving cables. As the length of the driving cables vary with the configuration variation of the segment, they are not constant for this kind of 2-DoF system and change in relation to the length of the driving cable; something which can be expressed as Eq. (4):

(4) $$k_{\text{cable},i}\left(l_{i}\right)=\frac{l_{0}}{l_{i}}k_{l_{o}},i=1,2,3$$

where $l_{0}$ is the initial length of the driving cable (related to the stiffness $k_{l_{o}}$), and $l_{i}$ is the variable length of the driving cable related to the configuration of the 2-DoF segment. The stiffness matrix of the compliant hinges, $K_{\text{hinge}}$, can be written with the stiffness matrix of the driving cables as Eq. (5).

(5) $$K_{\text{hinge}}=\left[\begin{array}{ccc} k_{\text{hinge},1} & 0 & 0\\ 0 & k_{\text{hinge},2} & 0\\ 0 & 0 & k_{\text{hinge},3} \end{array}\right]$$

where $k_{\text{hinge},1}$, $k_{\text{hinge},2}$ and $k_{\text{hinge},3}$ are the stiffness parameters of the three flexible hinges. A diamond shaped compliant hinge was selected in this paper to validate the proposed concept. To obtain the stiffness behaviour of the hinge, a software simulation was used to obtain the stiffness value under the given external load. Subsequently, the simulated result was substituted into the stiffness model of the 2-DoF segment developed in the previous sections, seen in Eq. (6).

(6) $$k_{\text{hinge},i}\left(l_{i}\right)=C_{2}h_{i}^{2}+C_{1}h_{i}+C_{0},i=1,2,3$$

where $C_{0}$, $C_{1}$ and $C_{2}$ are the coefficients of the equation (determined by the length and thickness of the compliant hinge) and $h_{i}$ is the length variation between the attached points in the upper and lower platforms respectively. As each section of the continuum robot is constructed by the same structured segments, the overall stiffness for the 2-DoF section with multiple segments can be written as Eq. (7):

(7) $$K_{\text{section}}=\frac{K_{\text{segment}}}{n_{\text{segment}}}$$

where $n_{\text{segment}}$ is the number of the segment for each section of the continuum robot and $K_{\text{segment}}$ is the stiffness of the 2-DoF segment. As the stiffness is imperative for keeping the static accuracy of the system under the external payloads, one section (with 14 segments) was selected as an example, and shown in Fig. 4. Under the given configuration of the continuum robot (phase angle φ and bending angle θ), the driving cables and flexible hinges were regarded as comprehensive springs (light blue line for the driving cable and bold blue line for flexible hinge) with different values.

**Fig. 4:**
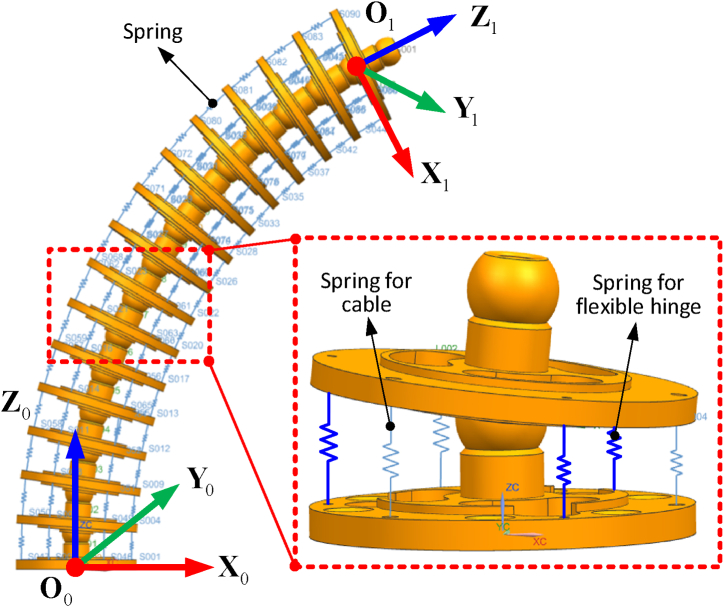
Stiffness model validation with the aid of software simulation.

**Fig. 5:**
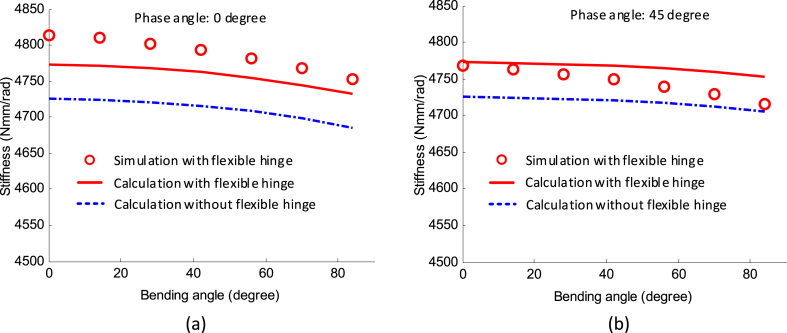
The stiffness model between the calculation and simulation on the example 2-DoF continuum robot: (a) and (b) represent phase angles of 0° and 45° respectively.

Three pairs of driving cables and flexible hinges are evenly spaced (120° interval) around the central ball joint, thus the stiffness behaviour of the system varies with the phase angle. Two sets of configurations were studied here where in each test, the phase angle is fixed (0° and 45°), while the bending angle is varied from 0° to 90° with an interval of 15°. The stiffness characteristic of the system at each testing point was calculated and simulated, as seen in Fig. 5, where Fig. 5(a) and (b) are the stiffness variations of the 2-DoF joint under the fixed phase angle (0° and 45°), respectively.

The stiffness of the robot matches the test points with high accuracy (i.e., 0.65% and 0.49% respectively), proving the correctness of the stiffness model developed in this paper. As an example, if the phase angle is 0°; with the increase of bending angle, the stiffness of the system decreases gradually from the simulation results (i.e., from 4814 N mm/rad to 4752 N mm/rad). When the phase angle is 45°, a similar trend of stiffness variation with 0° phase angle is obtained, although the value of stiffness is lower. Thus, it can be inferred that the stiffness of the robot is changing under the circular motion, i.e. the bending angle θ is constant, while the phase angle φ is changing.

## 4 Validations and performance tests

With the aid of the kinematic and stiffness models developed in previous sections, the input-output behaviour of the system (e.g., length of the driving cables and shape of the continuum robot, external loads & shape deformation) can be obtained. With these calculations, a set of experimental tests (e.g., kinematic improvement with the aid of flexible hinges were conducted with the structured prototype and control system.

### 4.1 System configuration

**Fig. 6:**
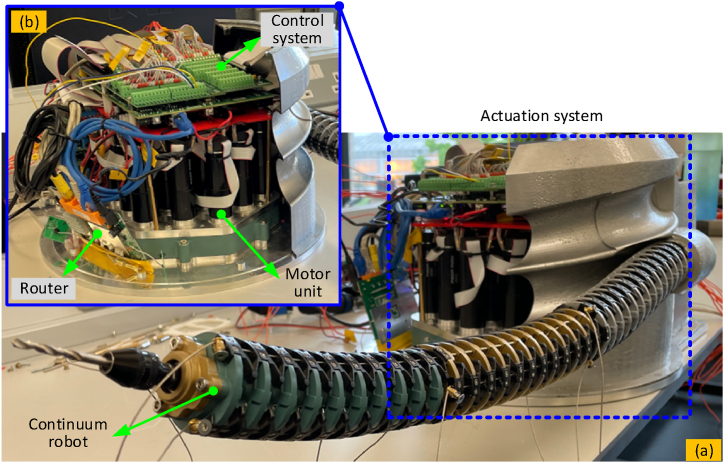
System configuration of the continuum robot with flexure hinges: (a) is the overall structure of the system; (b) is the actuation system for adjusting the length of the driving cables.

**Fig. 7:**
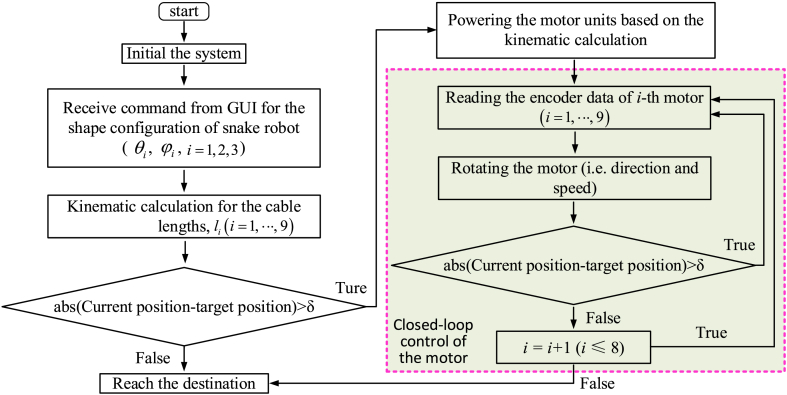
Flow chart for the kinematic control of the continuum robot (implemented with the LabView/FPGA hardwire).

Fig. 6 shows the structure of the dual-arm continuum robot, which is composed of a 6-DoF continuum arm, end effector, actuation system and hardware for the associated control system. Displacement closed-loop controllers were used to alter the shape of the manipulators and gripping motion; while for the milling piece a speed closed-loop controller was developed. In order to reduce the control period and improve the response speed of the system, a National Instruments sbRIO-9627 embedded controller has been used, while the GUI was developed with LabView software to regulate the real-time shape of the continuum robots. A flow chart for the kinematic control of the continuum robot (implemented with the LabView/FPGA hardwire) is shown in Fig. 7.

To improve the kinematic performance, the diameter of the manipulators and the width of the flexure hinges in section 1 were designed larger to improve the stiffness behaviour compared to the other two sections. The detailed structure parameters of the platform can be seen in Table 1.

**Table 1:** Structure parameters of the continuum robot.

Parameters	length (mm)	Diameter (mm)	Hinge width (mm)	Hinge thickness (mm)
Section-1	210	46	6	1
Section-2	180	43	5	1
Section-3	150	40	4	1

For each 2-DoF section, 3 Maxon motors (RE25 motors, GP26A gearboxes with a reduction ratio of 236, and an ENC16 encoder with 1024 pulses) were compactly arranged. Flexible steel cables covered by flexible spring tubing with higher compressing stiffness was also adopted. In addition, to fabricate the special shape of the flexible hinges for achieving the desired performance (i.e., higher rotational stiffness and low bending stiffness), the additive manufacturing method (machine type: Photocentric LC Magna, resolution: 137 μm, material: durable resin) was utilized here.

A control algorithm was developed based on the structured LabView FPGA control system (sbRIO-9627, LabView 2017). The closed-loop controller for the 9 motors of each manipulator runs within the FPGA hardware utilized. When the required phase and bending angle are received from the GUI, the kinematic calculation is first performed to obtain the appropriate length of each driving cable, before a closed-loop controller regulates the motors to the desired positions. During this stage, the phase angle and bending angle of each section will be adjusted to the right position.

### 4.2 Kinematic benefits aided by flexible hinges

**Fig. 8:**
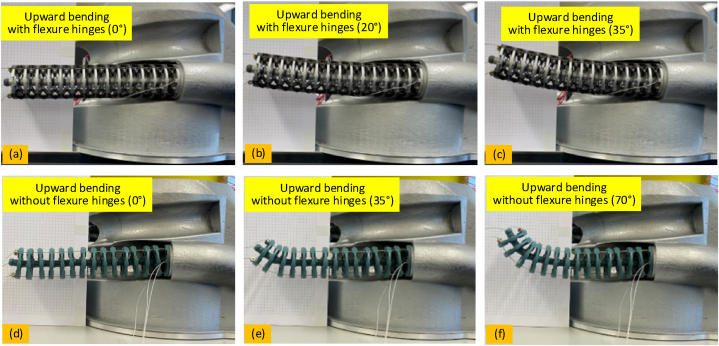
Experimental setup of the 2-DoF continuum robot for the kinematic accuracy testing: (a) to (c) are the motion tests of the 2-DoF continuum robot with the flexure hinges (bending angles are 0°, 25° and 35°, respectively); (d) to (f) are the motion tests of the 2-DoF continuum robot without the flexure hinges (bending angles are 0°, 35° and 70°, respectively).

In order to evaluate the kinematic performance of the manipulators, a 2-DoF continuum arm was selected as an example and configured as a cantilever beam. To capture the deviation, grid paper was utilized as the background for scaling the trajectory variation. To demonstrate the advantage of adding the flexure hinge to the manipulator, two kinds of robot arm were fabricated; with and without the flexure hinges. To perform the experiments, the robot was controlled by 3 motor units to achieve the upward and downward bending. Fig. 8 shows the images of the manipulator with different bending angles. Adding the flexure hinges to the robot may improve the stiffness performance but will likely reduce its bending range; thus, the maximum bending angles are different with and without flexure hinges (35° and 70°).

One section of the 6-DoF continuum robot (i.e., a 2-DoF continuum section formed by a set of modular 2-DoF segments), was selected as an example for validating the proposed model. Initially, the 2-DoF section was configured as a cantilever beam, with its base fixed while the displacement of the section under given external loads was measured, as seen in Fig. 8. Fig. 8 (a)–(f) are the shape changing of 2-DoF continuum section (i.e. with and without flexible hinges) under different bending angles. Grid paper (5 mm spacing) was utilized as the background for calibrating the shape of the section with a camera (to achieve the resolution of 0.2 mm in reality) placed in front of the test rig. The data received from the experiments is compared with the theoretical calculations.

**Fig. 9:**
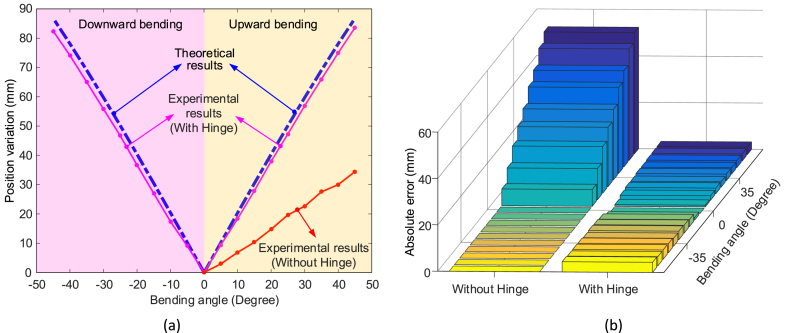
Positioning accuracy of the 2-DoF continuum robot under given bending degrees: (a) is the position deviation with different bending angles; (b) error between the experimental results and theoretical calculations.

By comparing the captured position with the calculated position under different degrees of bending, the positioning accuracy can be evaluated, as seen in Fig. 9.

It can be seen from Fig. 9(a) and (b) that the robot with a flexure hinge has an average positional accuracy error of 3.9 mm. The positioning accuracy of the 2-DoF continuum robot in the upward bending (average error: 3.4 mm) is higher than the downward bending (average error: 4.5 mm), mainly caused by the joint effect of gravity and the tension of the driving cables. For the 2-DoF continuum robot without the flexure hinges, the positioning accuracy is much poorer (average error: 34 mm). The reasons can be attributed as:

o No connections between the disks of the continuum robot, causing uncontrollable motion within them;
o Frictions in the ball joints causing the bending motion to occur closer to the tip sections;
o Flexure hinges can combine all the disks together, avoiding the uncontrollable motion of the disks;
o Tension in the driving cables will be increased to balance the material deformation of the flexure hinge;
o Reducing the backlash of the joints and slack of the driving cables.

## 5 Discussion

In this paper, a novel attempt has been made about introducing the compliant mechanism into the continuum robot for improving the kinematic performance. As a case study, the flexible hinges with diamond-shape were used to be equally spaced between the gaps of the backbone structures, aiming to adjust the performance of the entire system by their elastic deformation. With the studies of this paper (i.e., FEM simulation, stiffness modelling, control system, fabrication and experimental tests), it has been demonstrated that the developed concept is a useful way to improve the performance of the continuum robots.

## 6 Contributions

With the claimed novelties of introducing the compliant mechanism with continuum robot, the contributions of this paper can be summarized in the following aspects:

One contribution is the incorporation of the compliant mechanism into the continuum robot, enabling to adjusting the mechanical performance of the system (e.g., stiffness, backlash) in large scale. As a case study, diamond-shape leaf configuration of the flexible hinges was adopted and equally spaced between the gap of the backbone joints. With the aid of simulation and experimental tests, it has been demonstrated that the introduction of the flexible hinge is an effective way of improving the kinematic accuracy of the continuum robot (10 times). Besides the diamond-shape configuration of the flexible hinge used in case study, other alternative configurations (e.g., round-shape leaf, compliant rod, and hyperplastic rubber) are also possible for coping the challenges in different areas. Taking the compliant rod as the example, the flexible hinges can be replaced by the compliant rods with different parameters (e.g., length and diameter) and materials (e.g., Nitinol and ring steel) for achieving different performances.

Even some attempts have been made by introducing additional materials (e.g., meltable material [35]), mechanisms (e.g., compression spring [36], and inflatable unit [37]) for the continuum robot, further, to adjust the performance of the system, they are either to slow of the phase transition for changing their properties nor unstable/unchangeable of the claimed characters. For example, a kind of “stiffening sleeve”, which is made of heating elements and thermoplastics [35], was developed to tune the delivery of the continuum robot in different states (i.e., delivering: low stiffness, operating: high stiffness). However, the heating and cooling time are too slow for the real application. The compression spring was used to be coved on the sections of the continuum robot [36], aiming to improve the stiffness and reduce the backlash. However, the boundary conditions (e.g., physical contact with continuum robot, bending shape) of compression spring are too hard to be determined, resulting to the low accuracy of the model and unstable of the system. Further, the “inflatable unit” was developed to be combined with the continuum robot, aiming to adjust the stiffness of continuum robot rapidly. However, the difficulty of modelling and complexity of physical property bring challenges for the further application. To the best knowledge of author, the compliant mechanism, which has steady mechanical properties and rich configurations, is an ideal structure to be combined with the continuum robot.

The second contribution can be the modelling of the compliant mechanism with the continuum robots. Besides the great mechanical properties of the compliant mechanism itself, the rich modelling methods (e.g., Cosseart theory and compliant theory), advanced prototyping tools (e.g., wire electrical discharge machining, additive manufacturing) and various available materials (e.g., aluminium, ABS and resin) provide the possibility for developing new compliant mechanisms for the continuum robots. With the advanced manufacturing technologies, the real bionics design of the continuum robot (i.e., complicated compliant mechanisms with the similar functions of muscles are place around joints to achieve improved capability) can become possible. Then, the control and motion character of continuum robot will be no longer as current (i.e., highly relied on the length variation of the driving cables), and the more intelligent working pattern can be achieved (e.g., compliant mechanism as the muscle to control the motion of the joints).

### 6.1 Implications for practice

In recent years, continuum robots have been increasingly used in challenging environments (e.g., confined and complex spaces operation) due to their inherent dexterity, compact structure, large potential workspace and high environmental adaptability. However, most of the existing continuum robot are constructed by the modular backbone structures and actuated by long driving cables/tendons, resulting to the low control accuracy (mostly due to the slender and soft structure) for coping the high payload operations. Another challenge for the confined and complex spaces operation is the structure of the robot. Conventionally, single-arm continuum robot (e.g., engine combustor inspection [17,18] and minimally invasive surgery [38]) with specific end-effectors is largely used. However, more arms are needed when high criteria (e.g., high operation efficiency and improved operation dexterity) is required. This paper focus on solving the abovementioned challenges by proposing a new kind of dual-arm robotic system, aiming to improve the application of the continuum robot in the following aspects:

The first implication for practice of this paper is the development of a novel dual-arm robotic system, wherein two 6-DoF continuum, or snake, arms have been incorporated to perform cooperative tasks using specialized end-effectors (i.e. as an example, a rotary grinder is used on the end of the left arm, with a gripper on the right). This set-up has been configured to operate as a solid sample removal and retrieval system mounted to an underwater remotely operated vehicle (ROV) in challenging environments (e.g., decommissioning scenario). Besides combing the developed dual-arm robotic system with the ROV, the potential application can also be in the disaster assistance (e.g., by combing the robotic system with the quadrotor aircraft for the remote service) and telesurgery (e.g., by installing the dual-arm robot system at tip of the 6-DoF robot arm for achieving the increased workspace and improved operation accuracy).

The second implication for practise can be the incorporation of the compliant mechanism for overcoming the long-term shortcomings of the slender continuum robots. The first trail of combining the diamond-shape flexible hinge with a 3-section 6-DoF continuum robot has been demonstrated successfully in this paper. The results show that the kinematic performance of the continuum robot has been greatly improved. With the developed concept, a serial of trails of introducing the compliant mechanisms can be attempted in a wide range of slender continuum robots (e.g., small diameter: surgical [25,39] and inspection [18,37]; medium diameter: nuclear decommissioning [32,33]; large dimension: space station assembling [40] and electric car charging [41]) for improving the mechanical performance and miniaturing the dimension. Taking the surgical continuum robot as an example, the backbone structure with cable driving actuation strategy is normally used to change the shape of the robot. However, as only the super elastic rod/strip was normally used to provide the restrain force for the straight/initial shape, the effectiveness is limited for varying applications, especially when the mechanical performance of the system is required to be adjustable. By using the strategy of this paper, different structures of the compliant mechanism with varying dimensions can be designed to fit with the requirements (e.g., geometrical or mechanical) of the continuum robots, providing a great variety of choices for coping the varying demands.

The third implication for practise is the modelling of the compliant mechanism and multiple-DoFs manipulator. Conventionally, modelling the compliant mechanism alone involves lots of efforts for building kinetostatic model and computation resource for solving elastic deformation. When combining dozens of compliant mechanisms with continuum robot, the modelling complexity will increase exponentially, which become unrealistic for the real-time calculations and control. It can be seen from reference [32] that modelling and analysis of the spital branched flexure-hinge continuum robot is very complex, which is very hard for the general people to develop the model of themselves. To solve this challenge, a simplified model has been developed in this paper to reduce the complicity of building the stiffness model of the continuum robot but keep the modelling accuracy. Using the developed model, the stiffness behaviour of the continuum robot with different parameters can be studied, leading to the optimal parameter selection.

### 6.2 Limitations and future research

It has been demonstrated that introducing the compliant mechanism with continuum robot brings great advantage for improving the mechanical performance. However, designing an appropriate compliant mechanism is not an easy job, where lots of technologies and experience are needed, leading to the difficulty in design, prototyping and assembly. Due to the heavy labour and technology requirements, the limitations can be surmised as following:

1) The first is the selection of the suitable structure of compliant for the continuum robots. As the dimension and structure of the continuum robots are varying for different applications, the design of the compliant mechanisms is not an easy job. Overall, the design of the compliant mechanism should be within the existing limitations (e.g., space, motion characteristic and mechanical behaviour). Taking the compliant mechanism used in this paper as the example, the selected flexible hinge needs to meet the mechanical requirements of low bending stiffness, high rotation stiffness and 3-DoFs movements and geometrical requirements of miniature dimension, high elasticity and easy fabrication. At last, a kind of diamond-shape flexible hinge composed of four beams is selected to meet the aforementioned requirements. It can be seen from the listed example that rich experience and intensive technology is involved for selecting the appropriate compliant mechanism.
2) The second is fabrication of the compliant mechanism. As the compliant mechanisms will be filled within the gap of the continuum robot to aid the mechanical performances, which are similar with the muscles around the joints/spine of humans. For the large dimension (e.g., space station assembling [40] and electric car charging [41]) continuum robots, the design and material selection of the compliant mechanisms is easy as the space is sufficient. However, for the medium (e.g., nuclear decommissioning [32,33]) and miniature dimensions (e.g., surgical [25,39] and inspection [18,37]) continuum robots, it will becoming more difficult. Taking the surgical continuum robot as the example, the dimension of the robot is normally within 10 mm with limited shaping stroke (45° of each section), resulting to the small space for the flexible hinges. Currently, the super elastic NiTi rod of strip is normally adopted to provide the restraining energy. If we want to introduce the compliant mechanisms with the surgical continuum robot, the smart design (e.g., miniature structure with robust mechanical performance) and advanced prototyping technologies (e.g., 3D printing and WEDM) need to be involved.
3) The third is the modelling and control of the new hybrid continuum robot. It can be seen from the references that modelling the multiple-DoF continuum robot is difficult [14,19], let alone there are dozens of compliant mechanisms involved and each compliant mechanism is composed of several flexible beams [32]. In order to build the accuracy models (e.g., kinematic, stiffness, kinetostatic and dynamic) for building the control system (e.g., PCCD, Fuzzy logic, adaptative impedance control) for the continuum robots, large efforts are normally needed. Besides the modelling of continuum robots, modelling of the compliant mechanism involves much more technologies. As dozens of compliant mechanisms are normally spaced around the continuum robot, the compliant matrix of each compliant mechanism needs to be transformed to the global coordinate system for calculating the compliant matrix of the system. Using the similar principle, the global mass matrix and damping matrix can be obtained for establishing the dynamic equations of the system. After that, the control system can be developed based on the established dynamic equations. It can be seen from the listed steps that great mathematical calculations are involved for modelling and control of the hybrid continuum robot, which brings the challenges for the researcher with less knowledge of this area.

With the aforementioned challenges/limitations of introducing the compliant mechanism into the continuum robot, the future work will be targeted on them. The first research plan will be the design of the serial compliant mechanism for different dimension of the continuum robots; the second plan will be the prototyping of the compliant mechanism of different dimension using the cutting-edge technologies; the third plan will be the modelling and control of the hybrid continuum robot. The last will be promoting the application of the developed hybrid continuum robot for coping the tasks in challenges environments (e.g., surgical, electrical charger, underwater nuclear decommissioning).

## 7 Conclusions

A novel dual-manipulator continuum robot, constructed using modular 2-DoF backbones, has been developed to perform co-operative tasks. The first point of novelty is the combination of a compliant mechanism with a continuum robot to improve the kinematic performance of the conventional backbone-structure and high length-diameter ratio manipulators. Based on this idea, a class of novel compliant mechanisms have been developed (e.g., flexible hinges, compliant rods and hyper-elastic soft materials) to facilitate the adaption to different environments. With the experimental demonstration, the proposed method has shown to be a successful method of improving kinematic accuracy.

The second point of novelty concerns the new stiffness model of the proposed 6-DoF continuum robot, incorporating the models of the modular cable-driven 2-DoF parallel mechanism and the novel flexible hinges. With the developed kinematic algorithm, the configuration of the dual-arm continuum robot (bending angle and phase angle of each section) can be determined to perform the given task. Based on the stiffness model, it was found that the stiffness of the 6-DoF continuum robot is dependent on the driving cables, the flexible hinges, and the central rigid shaft. It was also found that the introduction of flexible hinges has the benefit of improving the stiffness isotropy of the 6-DoF continuum robot. These results have been validated with the aid of simulation.

With the experimental setup and closed-loop controller, the benefits of adding flexure hinges to the continuum robots has been demonstrated under a set of experiments. Comparing with the cases without the flexure hinges, it has been found that the kinematic accuracy found to have improved by a factor of 10 by adding flexible hinges. With the added flexible hinges, the controllable kinematic performance of continuum robot has been achieved (e.g., higher axial stiffness and lower bending stiffness). The cable driving metrology used in the design enables the COM of robot concentrated under the ROV, improving the stability of the entire system. All the electrical unites are located within the actuation box, ensuring the waterproofing capability of the system.

## CRediT authorship contribution statement

**N. Ma:** Formal analysis, Investigation, Methodology, Software, Writing – original draft. **D. Cheneler:** Conceptualization, Funding acquisition, Project administration, Supervision, Writing – review & editing. **S.D. Monk:** Conceptualization, Funding acquisition, Supervision, Writing – review & editing.

## Declaration of competing interest

The authors declare that they have no known competing financial interests or personal relationships that could have appeared to influence the work reported in this paper.
